# Relaxed natural selection contributes to global obesity increase more in males than in females due to more environmental modifications in female body mass

**DOI:** 10.1371/journal.pone.0199594

**Published:** 2018-07-18

**Authors:** Wenpeng You, Maciej Henneberg

**Affiliations:** 1 Adelaide Medical School, The University of Adelaide, Adelaide, South Australia, Australia; 2 Institute of Evolutionary Medicine, University of Zurich, Zurich, Switzerland; University of Lincoln, UNITED KINGDOM

## Abstract

**Objective:**

Relaxed natural selection, measured by Biological State Index (I_bs_), results in unfavourable genes/mutations accumulation in population. Obesity is partly heritable. We aim to examine and compare the effects of relaxed natural selection on male and female obesity prevalence.

**Methods:**

Data for 191 countries of the world were captured for this ecological study. Curvilinear regressions, bivariate and partial correlations, linear mixed models and multivariate linear regression analyses were used to examine the relationship between I_bs_ and sex-specific obesity prevalence. Per capita GDP, urbanization and caloric intake were controlled for as the confounding factors. Fisher r-to-z transformation, R^2^ increment in multivariate regression and F-test were used to compare the correlations.

**Results:**

Curvilinear regressions, bivariate and partial correlations (controlled for GDP, urbanization and calories) revealed that I_bs_ was significantly correlated to obesity prevalence of both sexes, but significantly stronger to male than to female obesity prevalence. Curvilinear regression models also showed strong correlations. Mixed linear models, with effects of GDP, urbanisation and caloric intake controlled for, showed that male and female average obesity prevalence rates were significantly higher in countries with greater I_bs_ value than their equivalents in countries with lower I_bs_. Between higher and lower I_bs_ countries, the gap of male obesity prevalence is 60% greater than the gap of female obesity prevalence. Stepwise multiple regression identified that I_bs_ was a significant predictor of obesity prevalence of both sexes. Multivariate regression showed that, adding I_bs_ as an obesity predictor, R^2^ increment in male model was significantly greater than in female model.

**Conclusions:**

Relaxed natural selection may drive males and females to accumulate metabolic faulty genes equally. Probably due to greater environmental, personal intervention in regulating female body mass, relaxed natural selection shows less contributing effects to female obesity prevalence than to male obesity prevalence. Gene therapy to prevent obesity may need to be also taken into account.

## Introduction

Being overweight was once considered a problem only of high-income countries, but now obesity prevalence is rising worldwide and affects both economically developed and developing countries [1]. Indeed, obesity and its sequelae are now so common that they are replacing undernutrition and infectious diseases as the most significant causes of ill-health [2]. Moreover, people considered overweight or obese have been subject to discrimination and prejudice [2, 3].

The Body Mass Index (BMI) is a common tool to determine body weight status. In WHO statistics [4–7], there are four body weight status definitions regarding individual adult’s BMI, i.e. obesity (BMI ≥30 kg/m^2^), overweight (BMI ≥ 25 kg/m^2^), normal (BMI > 18 kg/m^2^, but < 25 kg/m^2^) and underweight (BMI < 18 kg/m^2^).).

During the past three decades, extensive studies explored how non-genetic factors, such as excessive intake of energy, changes of food components, sedentary lifestyle and gut flora imbalance, contributed to body weight increase [8–19]. However, the conclusions of these studies are controversial and/or circumstantial. One of the underlying reasons might be that the important role of genetics in obesity [20] was not considered in these studies.

Natural selection is a key mechanism of evolution. However, its effects in shaping humans as a species may have been relaxed due to modern living conditions, and improved public health and medicine [21, 22]. Natural selection, together with mutations controls the frequency of genes, which determine human heritable traits. Population escaping from natural selection over successive generations may make the prevalence of their heritable traits subject to change due to the imbalance mutation/selection [20, 23]. A direct consequence of this process is that *de novo* mutations, including those affecting energy balance and metabolism, recently have accumulated at an unexpectedly significant pace [24–27]. Multiple mutations may be accumulated in genomes quickly, which influence the phenotype [28–30] after only a few generations.

The Biological State Index (I_bs_) measures the population level reproductive success [30–33]. Therefore, it can be used to measure the magnitude of relaxed natural selection at a population level. The I_bs_ calculation formula [31, 32] is:

$$I_{bs}=1-\;\sum_{x=0}^{x=\omega }\;d_{x}\;s_{x}$$

Where

d_x_ = the frequency of deaths at age x

s_x_ = the probability of not having completed fertility at age x

*ω*: the age at death of the oldest member of the group

The I_bs_ expresses an opportunity for an average individual born into a population to pass on genes to the next generation. The greater I_bs_ value is, the less opportunity for natural selection to act on the population through mortality because all individuals in that population survive to and through their reproductive period (15–50 years old). Further explanation and calculations of the I_bs_ are described in S1 Text and for the I_bs_ value of each country see S1 Table).

It was postulated that unfavourable genes may have been accumulating in human populations due to greatly relaxed natural selection in the past 100–150 years [30, 34–37]. This hypothesis has been tested in several studies [30, 34, 35, 38] and a very recent study argued that relaxation of natural selection may have been contributing to worldwide obesity prevalence due to accumulation of genes affecting metabolism in human populations [27]. The rationale of the study into the relationship between relaxed natural selection and obesity prevalence increase is as follows:

The probable effect of *de novo* mutations is detrimental. Each population has a segment who carry metabolism and energy balance fault genes. When members of this segment of a population participate in the reproduction, they may pass their metabolic fault genes into the next generation [27]. The frequency of metabolic fault genes will increase when a larger fraction of total population have opportunity to participate in reproduction under a given set of mortality conditions [31, 32]. However, only the contribution of relaxed natural selection to obesity prevalence in total population (both sexes) has been studied. No effects of relaxed natural selection on obesity prevalence separately in males and females were considered.

The topic of sex disparities in obesity remains largely underresearched, let alone addressed. From the perspective of total population at the country level, males and females in the next generation may share equal opportunities to inherit metabolic fault genes. However, worldwide, obesity is more prevalent in females (23.28%) than in males (15.89%) [7]. Studies of sex disparity in obesity considered differences in fat distribution [39, 40], body fat storage level [41–43], the role of parental investment [44] and the role of estrogen effect on obesity [45]. The interaction between genetic factors and sex in identical twins’ BMI has been reported [46, 47]. However, the effects of relaxed natural selection on obesity in different sexes at the population level have not been explored [48]. Due to obvious differences in body composition, fat distribution and hormonal regulation of metabolism, especially during pregnancy, lactation and post-partum periods, expression of different genes in males and females may be differently influencing energy balance of individuals.

Therefore, the objective of the present study was to evaluate and compare the role of the I_bs_ contribution to male and female obesity prevalence from a global perspective using country sex-specific obesity prevalence data.

## Materials and methods

Data are freely available from the websites of the UN agencies (WHO, the World Bank and FAO). Data sources were described in the manuscript and their specific URLs were indicated in the section of Reference. No ethical approval or written informed consent for participation was required.

### Data collection and selection

The WHO Global Health Observatory (GHO) data (2014) on estimated sex-specific obesity prevalence rates by country were obtained and used as the dependent variables [7]. The estimates of sex-specific prevalence rates of obesity are expressed as the percentage of population aged 18+ with BMI equal to or over 30 kg/m^2^.

In order to investigate sex differences longitudinally, we also extracted data on I_bs_ and on obesity prevalence rates of Australian females and males for the years 1976, 1981, 1986, 1991, 1996 and 2009 [49].

Country specific I_bs_ values were used as the independent variable. The I_bs_ calculation [31, 32] was based on the fertility data of each country published by United Nations in 2008 [50] and the mortality data of life tables (2009) published by World Health Organization (WHO) in 2012 [51]. These calculations were the same as in the previous study published by Budnik and Henneberg [27]. Calculations and interpretations of I_bs_ are further described in S1 Text. Australian longitudinal I_bs_ was calculated using data published by the Commonwealth Bureau of Census and Statistics. In terms of data availability and quality, for Australia we were only able to calculate the I_bs_ for the years of 1976, 1981, 1986, 1991, 1996 and 2009.

Urbanization (expressed as a percentage of the population living in urban areas in 2010) [52], mean caloric intake in 2011–2013 (expressed in grand total calories per capita per day) [53] and gross domestic product per capita (GDP, expressed in purchasing power parity in 2010 US dollars) [54] were considered and controlled for as the confounding factors. The reasons of selecting potential confounding factors include: 1) Due to more affordability of the increases in caloric intake [55], obesity has traditionally been considered as an affluence-related medical condition [56]. 2) Living in an urban setting leads to sedentary lifestyle (less physical activity) and poorer diets (more animal fats and sugar), which have been considered an important factor to increase the risk of obesity [1, 20, 57–59]. Urban living setting also mirrors the Western lifestyle.

We aligned the I_bs_ with prevalence rates of obesity in females and males and then matched them with GDP, caloric intake and urbanization. Country specific data for 191 countries were put in a uniform format. Each country was treated as an individual subject and all of their available information was analysed. For some countries an estimate of one or the other variable was missing, thus specific analyses have sample sizes varying from 168 to 191.

We also aligned Australian I_bs_ with obesity prevalence of Australian females and males for those years in which we were able to use the data for I_bs_ calculation in order to explore longitudinal trend.

Although the WHO Global Health Observatory (GHO) data repository (2014) [7] defined four levels of BMIs for males and females (obesity, overweight, normal and underweight), we only chose obesity prevalence rates in females and males for modelling, analysing and reporting the correlation and regression results because the results for obesity can be compared with the findings of the previous study conducted by Budnik and Henneberg [27].

### Data robusticity check

The diagnostic test was run to check if there was a problem of multicollinearity between the data we collected. All the tolerances were less than 0.20 and all the Variance Inflation Factors (VIF) were above 5, which indicates there was not multicollinearity issue [60] (S2 Table).

The Kolmogorov-Smirnov and Shapiro-Wilk tests were performed with SPSS to test the normality of distributions of variables used *(*Details see -S3 Table). All variables analysed here were not normally distributed, thus various data transformations as described below were performed for each method applied.

### Scatter plots

Worldwide, the relationships between the I_bs_ and each of the male and female obesity prevalence rates were explored and visualized in Microsoft Excel^®^ producing scatter plots. Scatter plots were also used to explore longitudinal correlations between the Australia-specific I_bs_ and Australian sex-specific obesity prevalence rates. The best fit trendlines were reported respectively.

### Curvilinear correlation analysis

Due to non-normal data distribution detected in the Kolmogorov-Smirnov and Shapiro-Wilk tests, partial correlation analysis was conducted using correlations of residuals, not the standard SPSS procedure. Logarithmic, exponential, power and polynomial regression models were fitted to the data and for each specific regression analysis the model producing the greatest fit by the least squares criterion (greatest coefficient of determination—R^2^) was applied. First, best curvilinear regression between GDP and sex-specific obesity prevalence has been obtained, then residuals of individual country points around that line were regressed on urbanisation. Residuals around the best regression of GDP-residuals on urbanisation were calculated (second-order residuals). These second-order residuals were regressed on the caloric intake and then residuals around this regression line calculated (third-order residuals). First order residuals (sex-specific obesity prevalence standardised on GDP), second order (sex-specific obesity prevalence standardised on GDP and urbanization) residuals and third order residuals (sex-specific obesity prevalence standardised on GDP, urbanization and caloric intake) were regressed on I_bs_ thus obtaining correlations of I_bs_ to sex-specific obesity prevalence corrected for effects of GDP only, GDP and urbanisation, and GDP, urbanisation and caloric intake respectively.

### Data analysis based on linear correlation models

When data were logarithmed, similar levels of Pearson r correlation and Spearman rho between all variables were obtained. This allows us to consider that the logged data distributions, though not normal, provide homoscedastic distributions as required for linear correlations. Therefore, the data analysis was performed in four steps:

1. Pearson correlation analysis was conducted to examine the strength and direction of the correlations between all variables.
  Considering the potentially abnormal data distribution, subsequently, nonparametric correlation analysis was performed with the same set of data to examine the magnitude of the potential differences between correlation coefficients between obesity prevalence and all variables calculated in Pearson and nonparametric correlation analyses.
2. Partial correlation analysis was performed to explore the independent linear correlations of I_bs_ to male and female obesity prevalence rates respectively while we controlled for GDP, urbanization and caloric intake.
  Fisher’s r-to-z transformation was conducted to assess significance level of differences between the Pearson’s r and partial correlation coefficients of I_bs_ to male and female obesity prevalence rates.
  Cohen’s *f*^2^ was used to calculate and report the “effect size” in the partial correlation analysis.
  Partial correlation analysis was also performed to explore the independent linear correlations of calories to male and female obesity prevalence rates respectively when we swapped I_bs_ as a predicting variable with calories as the potential confounder.
3. Standard multivariate linear regression (Enter) was calculated on log-transformed data to obtain and compare the Beta coefficients between sex-specific obesity prevalence and all independent variables, which included I_bs_, calories, GDP and urbanization.
  Standard multivariate linear regression (Stepwise) was performed to assess which non-I_bs_ predictor(s) made substantial contributions to variation in obesity, and then I_bs_ was added to the list of predictors to show improvement in model fits for males and females. The magnitudes of improvements in the two model fits were firstly compared with the absolute improvement values obtained from “the R^2^ improvement in male prevalence due to adding I_bs_” and “the R^2^ improvement in female prevalence due to adding I_bs_” respectively. F-test was used to compare and determine if there is significant difference between the magnitudes of the two improvements. We calculated the ratio (F value) of “the R^2^ improvement in male prevalence due to adding I_bs_” to “the R^2^ improvement in female prevalence due to adding I_bs_”. The calculated F value was compared with the value of p = 0.05 and p = 0.01 at degrees of freedom used in regression analyses.
4. The linear Mixed Model Analysis was conducted to summarise the results allowing us to intercept change at the country and regional levels after the data were nested within the WHO regions.
  For the application of mixed-effects models that are based on linear relations between variables, scales of GDP, urbanisation and caloric intake were transformed from interval to ordinal. Values of each variable were ordered/ ranked from the smallest to the largest, and the ranks standardised on numbers of observations because the numbers of countries for which values of GDP, Urbanisation and Caloric intake available differed somewhat (from 168 to 191). This way the rank of the country with the maximum value became 100 while the rank of the country with minimum value was 100*1/N that is a fractional number. This procedure produced rectangular distributions of all variables, thus these distributions became homoscedastic and as such acceptable for linear analyses. Averages of ordinally measured variables in the entire sample are 50.0 and thus their averages in variously grouped subsamples are easily interpretable. The mixed model with nested terms fixed and random effects using the Restricted Maximum Likelihood method of estimation was run.

The Insurance Hypothesis hypothesized that perceived food insecurity due to economic inequality may contribute to obesity in the economically developed world [61]. In order to make our study constructive, we located the country specific Gini index [62] as the measurement of the economic inequality to test its correlation with obesity in the economically developed world. It may take years for inequality to be exposed to humans before delayed obesity representation is noticeable. Therefore, we calculated the mean Gini index over a 5-year period (2008–2012) in each country to represent typical long-term exposure to the economic inequality. The Pearson’s r and non-parametric and partial correlation analyses were conducted to identify the correlation between Gini index and obesity prevalence. Pearson’s r, Spearman’s rho coefficient, partial correlation, the linear Mixed Model Analysis and multiple-linear regression analyses were conducted using SPSS v. 24. The statistical significance was set at the 0.05 level, but the significance levels at 0.01 and 0.001 were also reported.

## Results

**Fig 1:**
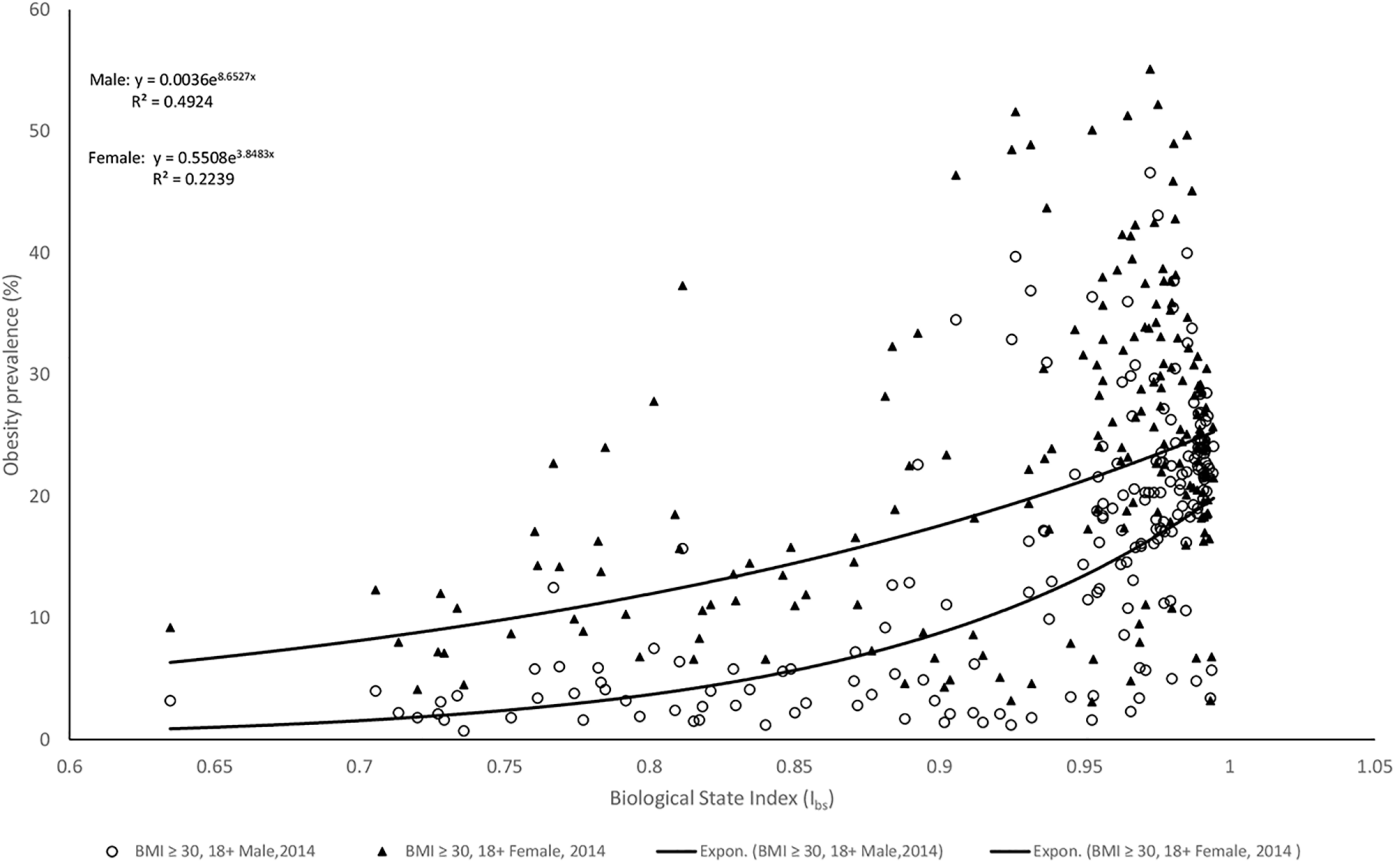
Relationships between I_bs_ and obesity prevalence estimates in males and females.

**Fig 2:**
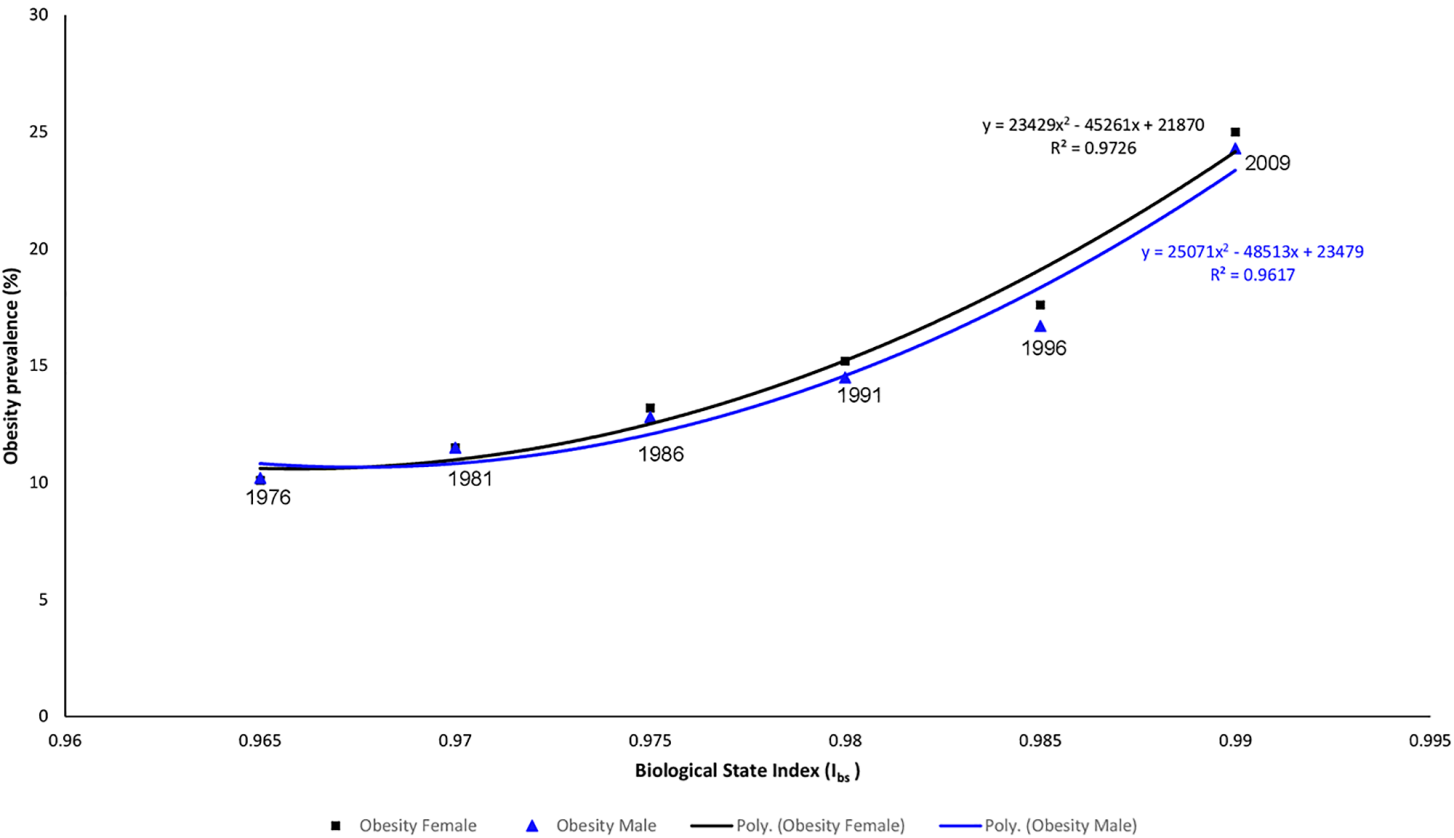
Longitudinal correlation between I_bs_ and sex-specific obesity prevalence in Australia.

Worldwide, I_bs_ was in strong and significant correlation (along exponential regression line) to both male obesity (r = 0.70, p<0.001) and female obesity (r = 0.47, p<0.001). Fisher r-to-z revealed that I_bs_ was in significantly stronger correlation to male obesity than to female obesity (z = 3.46, p<0.001) (Fig 1). Similar longitudinal trends were revealed between Australia-specific I_bs_ and Australian male and female obesity prevalence (Fig 2).

Curvilinear residual regressions revealed that I_bs_ was still significantly correlated to both male and female obesity prevalence when corrected for effects of GDP (r = 0.23, p<0.001 and r = 0.23 males and females respectively), GDP and urbanisation (r = 0.52, p<0.001 and r = 0.61, p<0.001 respectively), and GDP, urbanisation and caloric intake (r = 0.23, p<0.01 and r = 0.20, p<0.01 respectively). Fisher r-to-z transformation was conducted to compare the correlations between I_bs_ and male and female obesity prevalence respectively at the first, second and third order residuals, but there was no significant difference detected. This may suggest that relaxed natural selection contributes to male and female obesity equally regardless of the environmental intervention in regulating male and female body mass (Table 1).

**Table 1:** Curvilinear relationship between I_bs_ and male and female prevalence standardized on individual major obesity contributors in different combinations.

Prevalence %	Regression equation	r	n	Fisher r-to-z
Prevalence (Actual)				
Male	y = 0.0036e^8.6527x^	0.70	191	Z = 3.46, p = 0.0005
Female	y = 0.5508e^3.8483x^	0.47	191	
Prevalence Standardized on GDP				
Male	y = 0.1819x^0.4881^	0.34	184	Z = -1.26, p = 0.2077
Female	y = -360.53x^2^ + 639.48x − 278.31	0.23	184	
Prevalence Standardized on GDP and Urbanization				
Male	y = 694.64x^3^–1375.5x^2^ + 901.83x − 97.211	0.52	184	Z = -1.31, P = 0.1902
Female	y = 8369x^3^ − 19996x^2^ + 15903x − 4192.6	0.61	184	
Prevalence Standardized on GDP, Urbanization and Calories				
Male	y = -120.34x^3^ + 373.64x^2^–339.93x + 93.492	0.23	168	Z = 0.28, P = 0.775
Female	y = 2107.3x^3^–5332.6x^2^ + 4524.5x − 1292.5	0.20	168	

Sex specific obesity prevalence is the percentage of defined population segment with a body mass index (BMI) of no less than 30 kg/m^2^.

Data sources: Total calories data from the FAO’s FAOSTAT; BMI ≥30 data from the WHO Global Health Observatory; GDP data from the World Bank; Urbanization data from WHO. Biological State Index (I_bs_) was self-calculated with country specific fertility data published by the United Nations and the mortality data published by World Health Organization (WHO).

In Pearson correlation analysis, worldwide, I_bs_ was significantly correlated to both male (r = 0.692, p<0.001) and female (r = 0.470, p<0.001) obesity prevalence rates at levels similar to those for curvilinear regressions (Table 2). Similar values of correlation coefficients were observed in Spearman’s rho analysis as well indicating that log-transformation is sufficient to avoid substantial deviations from linear regressions in moment-product correlations (Table 2).

**Table 2:** Pearson r correlation (above the diagonal) Spearman rho (below the diagonal) between all variables*.

	Obesity %, Male	Obesity %, Female	I_bs_	Calories	GDP	Urbanization
Obesity %, Male	1	0.903**	0.692**	0.716**	0.761**	0.580**
Obesity %, Female	0.845**	1	0.470**	0.493**	0.517**	0.399**
I_bs_	0.667**	0.371**	1	0.639**	0.710**	0.513**
Calories	0.742**	0.451**	0.765**	1	0.759**	0.602**
GDP	0.758**	0.504**	0.866**	0.756**	1	0.672
Urbanization	0.583**	0.372**	0.666**	0.660**	0.736**	1

Pearson r and Spearman rho is reported. Number of countries included in the analysis ranges from 172 to 191.

*All correlations are significant at the 0.001 level (two-tailed).

Obesity % is percentage of defined population segment with a body mass index (BMI) of no less than 30 kg/m^2^.

Data sources: Total calories data from the FAO’s FAOSTAT; BMI ≥30 data from the WHO Global Health Observatory; GDP data from the World Bank; Urbanization data from WHO. Biological State Index (I_bs_) was self calculated with country specific fertility data published by the United Nations and the mortality data published by World Health Organization (WHO).

Fisher’s r-to-z transformation revealed that, in Pearson correlation analysis, I_bs_ was correlated to male obesity prevalence significantly stronger than to female obesity prevalence (z = 3.31, p<0.001) (Table 2).

Pearson correlation indicated that, worldwide, caloric intake was in significant correlation to both male (r = 0.0.716, p<0.001) and female (r = 0.493, p<0.001) obesity prevalence rates at levels similar to those for curvilinear regressions (Table 2). Fisher’s r-to-z transformation revealed that in Pearson correlation analysis, calories were correlated to male obesity prevalence significantly stronger than to female obesity prevalence (z = 3.3, p<0.001) (S4 Table).

Partial correlation analysis showed that, worldwide, the I_bs_ was still significantly correlated to the male and female obesity prevalence (r = 0.332, p<0.001 and r = 0.147, p<0.05 respectively) while we controlled for caloric intake, GDP and urbanization (Table 3). I_bs_ was, in partial correlation, significantly stronger to male obesity prevalence than to female obesity prevalence (z = 1.76, p<0.05) (Table 2).

**Table 3:** Correlation coefficients and Fisher’s r-to-z transformations of Pearson r and partial correlations between I_bs_ and male and female obesity prevalence.

Variable	Pearson correlation I_bs_				Partial Correlation I_bs_				
	n	r	p	Fisher’s r-to-z transformation	df	r	p	Effect Size	Fisher’s r-to-z transformation
Obesity %, Male	191	0.692	0.000	z = 3.31 p = 0.0005	163	0.332	0.000	0.124	z = 1.76 p = 0.039
Obesity %, Female	191	0.470	0.000		163	0.147	0.030	0.022	
GDP, USD 2010	184	0.710	0.000	-	-	-	-		-
Calories, mean 2011–13	172	0.639	0.000	-	-	-	-		-
Urbanization	191	0.513	0.000	-	-	-	-		-

Partial correlation (two-tailed) was run to examine the correlations between I_bs_ and male and female obesity prevalence respectively when GDP, Calories and urbanization were controlled for, but both the results were only reported.

-, Controlled variable.

Obesity % is percentage of defined population segment with a body mass index (BMI) of no less than 30 kg/m^2^.

Data sources: Total calories data from the FAO’s FAOSTAT; BMI ≥30 data from the WHO Global Health Observatory; GDP data from the World Bank; Urbanization data from WHO. Biological State Index (I_bs_) was self calculated with country specific fertility data published by the United Nations and the mortality data published by World Health Organization (WHO).

The effect size of I_bs_ on male obesity prevalence is 0.124, which is much greater than on female prevalence, 0.022 (Table 3).

Partial correlation analysis showed that, worldwide, caloric intake was still significantly correlated to the male obesity (r = 0.259, p<0.001), but not to female obesity prevalence (r = 0.140, p = 0.073) while we controlled for I_bs_, GDP and urbanization (S4 Table). However, the difference between the two correlation coefficients did not reach the significant level (z = 1.11, p = 0.134) (S4 Table).

The effect size of calories on male obesity prevalence is 0.072, which is much greater than on female prevalence, 0.020 (S4 Table).

Mixed linear models revealed that the male and female obesity prevalence rates were significantly different between WHO regions (F = 11.59, P<0.001 & F = 12.18, P<0.001 respectively) when GDP, urbanization and calories were controlled for. When the effects of GDP, urbanisation and calories were kept constant, mixed linear models revealed that male and female average obesity prevalence rates were significantly higher in countries with greater I_bs_ than their equivalents in countries with lower I_bs_, and that between higher and lower I_bs_ countries, the gap of male obesity prevalence (20.48%-9.54%) is 60% greater than the gap of female obesity prevalence (25.68%-18.85%) (Table 4 and Further details see S5 Table.

**Table 4:** Results of Mixed Model Analysis with the country specific data nested within WHO regions. Means of prevalence (%) of obesity (>30kg/m^2^) for males and females in countries with I_bs_ values above and below median are shown.

Males						
WHO Region	Countries with I_bs_ ≥0.9658			Countries with I_bs_ <0.9658		
	N	Mean	Std Deviation	N	Mean	Std Deviation
Africa	1	11.20	NA	39	5.19	3.97
Americas	24	20.72	4.59	11	14.95	4.71
Eastern Mediterranean	7	27.14	5.90	10	13.23	9.48
Europe	42	21.87	2.97	8	15.59	4.04
South-East Asia	3	4.70	1.18	6	2.78	1.35
West Pacific	9	14.46	11.68	8	17.76	14.28
Worldwide	86	20.48	6.60	82	9.54	8.23
Females						
WHO Region	Countries with I_bs_ ≥0.9658			Countries with I_bs_ <0.9658		
	N	Mean	Std Deviation	N	Mean	Std Deviation
Africa	1	24.30	NA	39	15.33	7.55
Americas	24	31.21	5.07	11	27.33	6.62
Eastern Mediterranean	7	39.60	4.38	10	23.02	13.26
Europe	42	22.99	4.09	8	21.39	3.56
South-East Asia	3	10.47	0.85	6	6.05	2.12
West Pacific	9	17.86	13.69	8	26.24	19.92
Worldwide	86	25.68	8.77	82	18.85	11.11

The mixed model with nested terms fixed and random effects using the Restricted Maximum Likelihood method of estimation was run.

Dependent Variable: BMI≥30 prevalence rates in Males and Females in 2014.

I_bs_ Med: Cutoff point of 0.9658.

NA: Not available

Multivariate regression model (Enter) revealed that I_bs_ was a significant (Beta = 0.287, p<0.001) predictor of male obesity prevalence when I_bs_, calories, GDP and urbanization were entered as the predicting variables. In contrast, I_bs_ was only a relatively weak and marginally significant (Beta = 0.180, p = 0.06) predictor of female obesity prevalence (Table 5).

**Table 5:** Results of enter and stepwise linear multivariate regression analyses to identify significant predictors of obesity prevalence in females and males.

**Enter**									
	Male obesity prevalence				Female obesity prevalence				
	I_bs_ excluded		I_bs_ included		I_bs_ excluded			I_bs_ included	
Variable	Beta	Sig.	Beta	Sig.	Variable	Beta	Sig.	Beta	Sig.
I_bs_	-	-	0.287	0.000	I_bs_	-	-	0.180	0.060
Calories	0.233	0.002	0.175	0.014	Calories	0.131	0.209	0.095	0.366
GDP	0.515	0.000	0.360	0.000	GDP	0.345	0.002	0.247	0.040
Urbanization	0.135	0.035	0.126	0.037	Urbanization	0.117	0.194	0.112	0.212
**Stepwise**									
	Male obesity prevalence				Female obesity prevalence				
	I_bs_ excluded		I_bs_ included		I_bs_ excluded			I_bs_ included	
Model	Variable	Adjusted R^2^	Variable	Adjusted R^2^	Model	Variable	Adjusted R^2^	Variable	Adjusted R^2^
1	GDP	0.606	GDP	0.606	1	GDP	0.268	GDP	0.268
2	Calories	0.635	I_bs_	0.657	2	Calories	Removed	I_bs_	0.284
3	Urbanization	0.642	Calories	0.673	3	Urbanization	Removed	Calories	Removed
4	-	-	Urbanization	0.680	4	-	-	Urbanization	Removed

Sex specific obesity prevalence is the percentage of defined population segment with a body mass index (BMI) of no less than 30 kg/m^2^.

All the selected predicting variables had the greatest influence on male and female obesity prevalence respectively at the significance level of p < 0.001.

Data sources: Total calories data from the FAO’s FAOSTAT; BMI ≥30 data from the WHO Global Health Observatory; GDP data from the World Bank; Urbanization data from WHO. Biological State Index (I_bs_) was self-calculated with country specific fertility data published by the United Nations and the mortality data published by World Health Organization (WHO).

Stepwise multivariate regression model results indicated that I_bs_ was, after GDP, the second strongest and significant predictor of both male and female obesity prevalence. The absolute improvement of R^2^ value due to adding I_bs_ in male model fit was 0.038 (from 0.642 to 0.680), which was more than double the absolute improvement value 0.016 (from 0.268 to 0.284) due to adding I_bs_ to female model fit (Table 5). This difference was significant (F value 2.375, p<0.01).

Interestingly, in the Stepwise multivariate regression model, caloric intake was one of the significant predictors of male obesity prevalence rate, but was not selected as one of the variables which had the greatest influence on female obesity prevalence (Table 5).

Table 6 represented the correlation between Gini index and obesity prevalence. No strong or significant correlation between Gini index and male or female obesity prevalence was established in the Pearson’s r and non-parametric and partial correlation analyses (Table 6).

**Table 6:** Correlation between Gini index and obesity prevalence in the developed world.

	Pearson r			Spearman’s rho			Partial Correlation		
	r	p	n	r	p	n	r	p	Df
Male obesity prevalence	-0.039	0.837	30	-0.063	0.742	30	-0.247	0.223	24
Female obesity prevalence	0.086	0.652	30	0.226	0.229	30	-0.124	0.548	24
Biological State Index (I_bs_)	-0.272	0.145	30	-0.083	0.661	30	-	-	-
Calories	0.162	0.393	30	0.237	0.208	30	-	-	-
GDP	-0.078	0.681	30	-0.078	0.682	30	-	-	-
Urbanization	-0.190	0.314	30	-0.033	0.862	30	-	-	-

Sex specific obesity prevalence is the percentage of defined population segment with a body mass index (BMI) of no less than 30 kg/m^2^.

Data sources: Total calories data from the FAO’s FAOSTAT; BMI ≥30 data from the WHO Global Health Observatory; GDP data from the World Bank; Urbanization data from WHO. Biological State Index (I_bs_) was self-calculated with country specific fertility data published by the United Nations and the mortality data published by World Health Organization (WHO). Gini index from the World Bank.

## Discussion

The worldwide trend of increased obesity prevalence is a multi-factorial phenomenon with major contributions from the environmental factors and the genetics. By assessing the data from 191 countries on the prevalence rates of the sex-specific obesity, we have shown that, globally, countries which had greater value of the I_bs_ (less opportunity for natural selection) have greater obesity prevalence rates in both males and females. These trends remained independent of the commonly considered drives (total caloric intake, urbanization and GDP) of obesity.

Through relaxing natural selection, the advanced technology and medicine may have had the dual role on obesity in the past 100–150 years [30, 35]. They may have made modern humans “well adapted” to their environment [30, 33, 35]. Meanwhile, they may have allowed the deleterious genes/mutations to accumulate in human population [30, 33, 38] as the population carrying deleterious genes/mutations would be able to reproduce and pass on the inheritable genes/mutations to their next generation. This hypothesis has been tested on population obesity and thinness by Budnik and Henneberg [27] and by Staub and Henneberg et al [63]. Theoretically, metabolic faulty genes/mutations may be cumulative in females and males at the same pace in the process of relaxation of natural selection. The aetiology of how relaxed natural selection (I_bs_) contributes to obesity in males and females has been discussed in detail elsewhere [27, 63].

The other important and new finding in this study was that, statistically, in Pearson analysis, the I_bs_ was in significantly stronger correlation to male obesity prevalence (r = 0.692, p<0.001) than to female obesity prevalence (r = 0.470, p<0.001). Fisher’s r-to-z transformation revealed that this difference was significant (z = 3.31, p<0.001). This relationship remained when GDP, caloric intake and urbanization were controlled for in partial analysis. The I_bs_ correlated to male obesity prevalence (r = 0.332, p<0.001) significantly stronger than to female obesity prevalence (r = 0.147, p<0.05). Fisher’s r-to-z transformation also revealed that this difference was significant (z = 1.76, p<0.05).

Considering the equal opportunities to inherit and accumulate metabolic faulty genes/mutations in males and females, the I_bs_ should be correlated to the obesity prevalence equally in females and males. The significantly weaker relationship between I_bs_ and female obesity prevalence in our analyses may indicate that the effects of relaxed natural selection on obesity are moderated by environmental factors more in females than in males. In other words, the same magnitude of faulty metabolic genes/mutations accumulation in males and females does not produce the same phenotypic outcomes at population level (i.e., different obesity prevalence rates). Multiple environmental factors that may influence the female obesity prevalence in different countries or regions may explain the disparity of obesity prevalence in males and females. Below is the listing of some possible environmental factors which may weaken effect of I_bs_ on obesity prevalence among females:

1. Fertility is a nutritionally expensive process for women due to gestation and lactation [44]. Therefore, women at reproductive age have been especially susceptible to excessive fat storage from the perspective of evolutionary biology [44]. Birth rates are low in developed countries, but high in developing countries [64, 65]. Nutrition stored in the form of fatness in females of developed countries, which is supposed to be used for successful reproduction, is simply kept without use, which increases body weight of females in the developed world. This is a result of conscious birth control, unrelated to genetic variation.
2. Toward the end of the 20^th^ century, there has been a transition away from agricultural labor (both for production and subsistence) to wage labor in many developing countries. This transition has decreased the physical activity of women more than men [66, 67].
3. Low birth rates in developed world [65] may make females exposed to more oestrogen due to more menstrual cycles, which may increase fat storage [45, 68].
4. Importantly, worldwide, different sociocultural beliefs and practices may also affect female disparities in excessive weight gain [69–73]. In general, females are socialized to be more appearance-focused than males [69], and they tend to adjust their personal environment (including working, eating, dieting and exercising) to control their body weight gain. Therefore, females’ natural genetic endowment for body weight may have less influence on their actual phenotype. For instance, females have been overprotected and, due to cultural [74] or religious [75] barriers, cannot publicly participate in physical activity in conservative societies, such as in the developing countries in the Middle East [76] and North Africa [77] region and the economically developed countries of Oman [78, 79], Kuwait [80, 81], and Saudi Arabia [82]. On the other hand, in the “Western” countries, the female body ideal has been that of a thin person for the last 50 years.

In terms of the relationship between caloric intake and male and female obesity, our analysis results indicated that: 1) caloric intake was in significantly stronger (z = 3.31, p<0.001) correlation to male obesity (0.716, p<0.001) than to female obesity (r = 0.493, p>0.001) in bivariate correlation analysis. 2) When we controlled for GDP, urbanization and I_bs_, caloric intake was in stronger (but not reached significant level) correlation to male obesity (r = 0.259, p<0.001) than to female obesity (r = 0.140, p = 0.073) in partial correlation analysis. 3) In Stepwise linear regression model, caloric intake was selected as one of the significant predictors for male prevalence (Adjusted R^2^ = 0.673, p<0.001), but not for female obesity. These controversial results may explain the finding in the study conducted by Budnik and Henneberg that caloric intake was not one of the variables which had the greatest influence on obesity in total population [27]. The underlying reason of these controversial results may be that females are much more appearance-focused than males. It may suggest that females’ greater focus on appearance may modify their natural genetic endowment for body weight more than males. It seems that caloric intake alone, without taking into account other factors that influence energy balance, for instance, food composition [18, 83, 84], may not be a strong predictor of obesity levels. A separate new study to further investigate the sex disparity in obesity between caloric intake and male and female obesity prevalence may be worth conducting.

Interestingly, female obesity prevalence, in general, correlates less strongly with country-characteristic variables than male obesity (Table 2). It may reflect results of industrialisation and economic situation because the ratio of male to female obesity per country shows linear and strong correlation (r = 0.77, P<0.001) to GDP with male/female ratios being less than one in countries with GDP below about 25,000 USD and above 1 in wealthier countries [45] There may be two reasons: 1) Males are exposed to environmental estrogen-like substances, such as dietary xenoestrogens (estrogens present in environment that may be ingested by people with food or water) associated with affluence [45]. 2) In the “Western” countries, the female body idol has been that of a thin person for the last 50 years. Therefore, individual female in the Western world may concern her body mass to be driven by requirements of fashion to a larger extent than those of males.

From evolutionary perspective, there are several hypotheses proposed to explain the modern obesity issue. The “thrifty gene” hypothesis proposed that obesity predisposing genes were advantageous in hunting and gathering period, but detrimental in the modern world [85]. As an alternative to the thrifty gene hypothesis [86] [87], the “drifty gene” theory postulated that obesogenic energy-efficient genes favoring fat storage are present in modern humans because of the removal of predative natural selection pressures [87, 88]. Sellayah *et al*. believed that poor adaptation to environmental factors in modern humans may contribute to obesity after they emigrated from Africa around 70,000 years ago [89], if they ever did.

All these three hypotheses would require thousands of years evolution to slowly accumulate the genetic background of obesity. This makes these hypotheses irrelevant to our study as we are advancing a hypothesis that metabolic faults caused by mutations have been accumulating in human populations at previously unexpected speed [24–26, 30, 90] because natural selection has been relaxed sharply in the last 100–150 years [27, 30, 38] [33]. Our hypothesis also implies that modern humans may not be naturally well adapted to the current environment because the advanced technologies and medical services may have been artificially modifying their metabolic processes [27, 30, 33, 38]. This implication may not be inferred from the other three hypotheses.

Supported by the drifty gene theory, the Insurance Hypothesis (IH) advanced that food insecurity, instead of food abundance, may contribute to obesity [61], and it was found that in high income populations (also called Western countries in common practice), perceived food insecurity due to social inequalities was associated more with obesity prevalence among adult women than men [61]. Gini index, a common measurement of economic inequality, however, did not show correlation with obesity.

In this study, the curvilinear correlation was applied as the Kolmogorov-Smirnov and Shapiro-Wilk tests detected that the data distributions are not normal. It is revealed that I_bs_ is correlated to sex-specific obesity prevalence residuals which were obtained by removing the contributing effects of non-genetic (environmental) factors from obesity prevalence, but there is no significant difference between the two correlations within the 1^st^, 2^nd^ and 3^rd^ order residuals. This finding may complement our hypothesis because this may imply that relaxed natural selection has increased the frequencies of obesity genes/mutations in males and females equally.

Population-based prevention strategies targeting ‘‘obesogenic” environments have been advocated and adopted as a public health approach [91, 92]. However, unfortunately, no country has achieved their expected results in the past 30 years [93]. The process of natural selection reduction which has driven the accumulation of the energy balance and metabolic faulty genes/mutations in human populations may partially explain this phenomenon [30]. Random mutations are as likely to affect metabolism to produce too much adipose tissue as not to and reduce body mass excessively [27]. There is, however, a simple imbalance between the two directions of metabolic faults—body mass of a living human being cannot be reduced much below a certain level determined by the weight of musculo-skeletal, circulatory, urinary, reproductive, nervous and integumentary systems, while it can be doubled, tripled, or even, perhaps, quadrupled by increasing the amount of adipose and muscle tissue. This imbalance produces, on average, increase in body mass and in prevalence of obesity over that of underweight.

Several generations of people in Europe and North America have had the access to advanced medical care earlier and easier than those from the developing areas, such as Africa and Asia. This may be one of the reasons that obesity has become a noticeable pressing issue much earlier in the developed regions. For instance, Olshansky *et al*. reported that the life expectancy in the USA may be reduced if obesity prevalence keeps rising in the future [94].

Several limitations in this study need to be acknowledged:

First, the relationship between I_bs_ and obesity prevalence reported here only shows coincidence, not causality.

Second, we could only demonstrate the relationship between the I_bs_ and the obesity prevalence rate at country/population level, rather than at the individual level because both data analysed [31, 32] and the evolutionary approach [23] are population based.

Third, although we controlled for total caloric intake as one of the potential confounders, due to the different diet/nutrition patterns between males and females, the different contribution of nutrition/diet to obesity levels in males and females should have been considered. However, we could not obtain the data for correlation analysis in this study.

Fourth, the changes in the genomes of human populations may be too slow to fully explain the increasing obesity prevalence. Obesity is the result of an unfavourable interaction between our genomes and our current environment which might play more important role in developing obesity in some circumstances.

Fifth, this study analysed the data across 191 countries. However, the results cannot be complemented by the longitudinal data analysis in individual countries, with exception of Australia and Poland [27] due to the fact that obesity only has been an issue in the last few decades. We could not access the combined obesity and I_bs_ data which are older than 30 years.

Finally, the female complexities, adaptation for fertility [44], more oestrogen [68] and double X chromosomes in cells [95] may have confounded our analysis of correlation of the I_bs_ to female obesity prevalence, but we could not obtain data to reduce or avoid such confounding effects.

The natural selection has been universally relaxed, and this trend continues worldwide, the medical services keep improving quickly. Recent advances in genome editing have made gene therapy possible to knock out the genes/mutations in relation to obesity [96]. For instance, Gendicine and Glybera have been used for treatment of head and neck squamous cell carcinoma [97] and lipoprotein lipase deficiency [98] respectively. The obesity related genes/mutations accumulation in human populations through the process of reduction of natural selection may become more and more imperative. Advances in our knowledge of the molecular basis of obesity and obesity-associated diseases, and development of gene therapy may offer an alternative long-term treatment modality in the near future.

## Conclusions

Recently accumulated high frequency of genes related to metabolic faults in human populations may be one of the important contributors to the increasing prevalence of obesity worldwide. The relaxed natural selection may have accumulated metabolic faulty genes in both males and females over successive generations. Relaxed natural selection affecting less female obesity prevalence than its male equivalent may be attributable to female-specific physiological mechanisms and various socio-cultural practices. Public health approaches based solely on consideration of energy balance to develop population-based strategies for the prevention of excess weight gain may not be able to achieve expected results. Genetics may need to be also taken into account.

## Supporting information

S1 Table Ibs values for 191 countries. (DOCX)

S2 Table Multicollinearity (diagnostic tests) among the predictors. (DOCX)

S3 Table Tests of normality of distributions of studied variables. (DOCX)

S4 Table Correlation coefficients and Fisher’s r-to-z transformations of Pearson r and partial correlations between calories and female and male obesity prevalence. (DOCX)

S5 Table Results of Mixed Model Analysis with the data nested within the WHO regions and country groupings with greater and lower median of I_bs_. (XLSX)

S1 Text Calculation and significance of Biological State Index (Ibs). (DOCX)
