# Supplementary material for: Relaxed natural selection contributes to global obesity increase more in males than in females due to more environmental modifications in female body mass
Source: PLoS One. 2018 Jul 18;13(7):e0199594. doi: 10.1371/journal.pone.0199594 (PMC6051589; doi:10.1371/journal.pone.0199594)
Supplement: S1 Table — (DOCX) [file pone.0199594.s001.docx]

S1 Table: I_bs_ values for 191 countries [1]

| Country | I_bs_ Value | Country | I_bs_ Value | Country | I_bs_ Value | Country | I_bs_ Value |
| --- | --- | --- | --- | --- | --- | --- | --- |
| Afghanistan | 0.71993 | Dominican Republic | 0.95615 | Luxembourg | 0.99236 | Singapore | 0.99372 |
| Albania | 0.97509 | Ecuador | 0.96240 | Macedonia, FYR | 0.98626 | Slovakia | 0.98890 |
| Algeria | 0.95412 | Egypt | 0.97077 | Madagascar | 0.91151 | Slovenia | 0.98959 |
| Andorra | 0.99196 | El Salvador | 0.96938 | Malawi | 0.77708 | Solomon Islands | 0.94653 |
| Angola | 0.76887 | Equatorial Guinea | 0.76681 | Malaysia | 0.98480 | Somalia | 0.72700 |
| Antigua & Barbuda | 0.97687 | Eritrea | 0.91484 | Maldives | 0.97995 | South Africa | 0.81135 |
| Argentina | 0.97630 | Estonia | 0.98915 | Mali | 0.77401 | Spain | 0.99165 |
| Armenia | 0.97639 | Ethiopia | 0.81512 | Malta | 0.99007 | Sri Lanka | 0.96877 |
| Australia | 0.98989 | Fiji | 0.96733 | Marshall Islands | 0.93130 | St. Kitts and Nevis | 0.97952 |
| Austria | 0.99094 | Finland | 0.99088 | Mauritania | 0.82887 | St. Lucia | 0.97066 |
| Azerbaijan | 0.95952 | France | 0.99155 | Mauritius | 0.97732 | St. Vincent & the Grenadines | 0.97710 |
| Bahamas | 0.97392 | Gabon | 0.88953 | Mexico | 0.97618 | Sudan | 0.82098 |
| Bahrain | 0.98112 | Gambia | 0.84868 | Micronesia, Fed. Sts. | 0.93688 | Suriname | 0.95629 |
| Bangladesh | 0.92070 | Georgia | 0.96274 | Moldova | 0.97951 | Swaziland | 0.80146 |
| Barbados | 0.98129 | Germany | 0.99209 | Mongolia | 0.96440 | Sweden | 0.99242 |
| Belarus | 0.98294 | Ghana | 0.88456 | Montenegro | 0.98737 | Switzerland | 0.99290 |
| Belgium | 0.99016 | Greece | 0.99101 | Morocco | 0.95492 | Syrian Arab Republic | 0.97597 |
| Belize | 0.96940 | Grenada | 0.97452 | Mozambique | 0.75217 | Tajikistan | 0.93765 |
| Benin | 0.83445 | Guatemala | 0.93855 | Myanmar | 0.90156 | Tanzania | 0.82970 |
| Bhutan | 0.89433 | Guinea | 0.79173 | Namibia | 0.88120 | Thailand | 0.97093 |
| Bolivia | 0.93065 | Guinea-Bissau | 0.73362 | Nauru | 0.92612 | Timor-Leste | 0.92455 |
| Bosnia & Herzegovina | 0.93065 | Guyana | 0.94943 | Nepal | 0.93168 | Togo | 0.85388 |
| Botswana | 0.88361 | Haiti | 0.87088 | Netherlands | 0.99118 | Tonga | 0.95252 |
| Brazil | 0.97459 | Honduras | 0.95493 | New Zealand | 0.98754 | Trinidad & Tobago | 0.95607 |
| Brunei Darussalam | 0.98497 | Hungary | 0.98901 | Nicaragua | 0.96483 | Tunisia | 0.97206 |
| Bulgaria | 0.98359 | Iceland | 0.99434 | Niger | 0.79660 | Turkey | 0.97458 |
| Burkina Faso | 0.63476 | India | 0.89826 | Nigeria | 0.78223 | Turkmenistan | 0.93614 |
| Burundi | 0.73592 | Indonesia | 0.94507 | Niue | 0.98062 | Tuvalu | 0.90554 |
| Cambodia | 0.88794 | Iran | 0.96320 | Norway | 0.99131 | Uganda | 0.81707 |
| Cameroon | 0.76039 | Iraq | 0.93584 | Oman | 0.97720 | Ukraine | 0.97745 |
| Canada | 0.98911 | Ireland | 0.98989 | Pakistan | 0.87655 | United Arab Emirates | 0.98692 |
| Cape Verde | 0.96350 | Israel | 0.99116 | Palau | 0.97515 | United Kingdom | 0.98988 |
| Central African Republic | 0.71339 | Italy | 0.99201 | Panama | 0.96705 | Uruguay | 0.97986 |
| Chad | 0.70549 | Jamaica | 0.95615 | Papua New Guinea | 0.89240 | USA | 0.98535 |
| Chile | 0.98569 | Japan | 0.99324 | Paraguay | 0.96662 | Uzbekistan | 0.95421 |
| China | 0.96889 | Jordan | 0.96117 | Peru | 0.96741 | Vanuatu | 0.96286 |
| Colombia | 0.97377 | Kazakhstan | 0.95456 | Philippines | 0.95294 | Venezuela | 0.97379 |
| Comoros | 0.85001 | Kenya | 0.87165 | Poland | 0.98881 | Viet Nam | 0.96584 |
| Congo | 0.81029 | Kiribati | 0.92477 | Portugal | 0.99074 | Yemen | 0.90227 |
| Congo, Dem. Rep. | 0.72917 | Korea, Dem. Rep. | 0.95241 | Qatar | 0.98518 | Zambia | 0.76135 |
| Cook Islands | 0.97249 | Korea, Rep. | 0.98835 | Romania | 0.98266 | Zimbabwe | 0.80874 |
| Costa Rica | 0.98362 | Kuwait | 0.98038 | Russian Federation | 0.97611 |  |  |
| Côte d'Ivoire | 0.78317 | Kyrgyzstan | 0.95104 | Rwanda | 0.83990 |  |  |
| Croatia | 0.98889 | Lao PDR | 0.90351 | Samoa | 0.96475 |  |  |
| Cuba | 0.98887 | Latvia | 0.98514 | Sao Tome and Principe | 0.91189 |  |  |
| Cyprus | 0.99406 | Lebanon | 0.97970 | Saudi Arabia | 0.96580 |  |  |
| Czech Republic | 0.99174 | Lesotho | 0.78464 | Senegal | 0.87041 |  |  |
| Denmark | 0.99132 | Liberia | 0.81816 | Serbia | 0.98868 |  |  |
| Djibouti | 0.84585 | Libya | 0.96632 | Seychelles | 0.97996 |  |  |
| Dominica | 0.98204 | Lithuania | 0.98782 | Sierra Leone | 0.72789 |  |  |

1. Budnik, A. and M. Henneberg, *Worldwide Increase of Obesity is Related to the Reduced Opportunity for Natural Selection.* PLOS One, 2017. **12**(1).
