# Supplementary material for: Relaxed natural selection contributes to global obesity increase more in males than in females due to more environmental modifications in female body mass
Source: PLoS One. 2018 Jul 18;13(7):e0199594. doi: 10.1371/journal.pone.0199594 (PMC6051589; doi:10.1371/journal.pone.0199594)
Supplement: S2 Table — (DOCX) [file pone.0199594.s002.docx]

S2 Table: Multicollinearity (diagnostic tests) among the predictors

Table AF 2-1: Multicollinearity tests of male and female obesity prevalence to other predictors

| Independent Variables | Tolerance | VIF |  | Independent Variables | Tolerance | VIF |
| --- | --- | --- | --- | --- | --- | --- |
| Urbanization | 0.535 | 1.869 |  | Urbanization | 0.535 | 1.869 |
| I_bs_ | 0.471 | 2.124 |  | I_bs_ | 0.471 | 2.124 |
| Calories | 0.386 | 2.590 |  | Calories | 0.386 | 2.590 |
| GDP | 0.299 | 3.344 |  | GDP | 0.299 | 3.344 |

Table AF 2-2: Multicollinearity tests of calories to other predictors in male and female samples respectively

| Independent Variables | Tolerance | VIF |  | Independent Variables | Tolerance | VIF |
| --- | --- | --- | --- | --- | --- | --- |
| GDP | 0.304 | 3.290 |  | GDP | 0.358 | 2.793 |
| Urbanization | 0.542 | 1.846 |  | BMI ≥ 30, Female | 0.697 | 1.434 |
| BMI ≥ 30, Male | 0.324 | 3.084 |  | Urbanization | 0.558 | 1.791 |
| I_bs_ | 0.424 | 2.360 |  | I_bs_ | 0.474 | 2.108 |

Table AF 2-3: Multicollinearity tests of GDP to other predictors in male and female samples respectively

| Independent Variables | Tolerance | VIF |  | Independent Variables | Tolerance | VIF |
| --- | --- | --- | --- | --- | --- | --- |
| Urbanization | 0.553 | 1.809 |  | BMI ≥ 30, Female | 0.712 | 1.404 |
| BMI ≥ 30, Male | 0.351 | 2.848 |  | Urbanization | 0.580 | 1.723 |
| I_bs_ | 0.453 | 2.207 |  | I_bs_ | 0.548 | 1.826 |
| Calories | 0.425 | 2.353 |  | Calories | 0.472 | 2.119 |

Table AF 2-4: Multicollinearity tests of I_bs_ to other predictors in male and female samples respectively

| Independent Variables | Tolerance | VIF |  | Independent Variables | Tolerance | VIF |
| --- | --- | --- | --- | --- | --- | --- |
| Calories | 0.376 | 2.659 |  | Calories | 0.395 | 2.529 |
| GDP | 0.288 | 3.476 |  | GDP | 0.346 | 2.888 |
| Urbanization | 0.521 | 1.918 |  | BMI ≥ 30, Female | 0.709 | 1.410 |
| BMI ≥ 30, Male | 0.351 | 2.848 |  | Urbanization | 0.530 | 1.886 |

Table AF 2-5: Multicollinearity tests of urbanization to other predictors in male and female samples respectively

| Independent Variables | Tolerance | VIF |  | Independent Variables | Tolerance | VIF |
| --- | --- | --- | --- | --- | --- | --- |
| I_bs_ | 0.419 | 2.385 |  | I_bs_ | 0.461 | 2.170 |
| BMI ≥ 30, Male | 0.321 | 3.116 |  | Calories | 0.405 | 2.472 |
| Calories | 0.387 | 2.586 |  | GDP | 0.319 | 3.134 |
| GDP | 0.282 | 3.542 |  | BMI ≥ 30, Female | 0.701 | 1.427 |

**Note for the above 5 tables:**

A tolerance of less than 0.20 or a VIF of above 5 indicates a multicollinearity problem.

Sex specific obesity prevalence is the percentage of defined population segment with a body mass index (BMI) of no less than 30 kg/m^2^.

Data sources: Total calories data from the FAO’s FAOSTAT; BMI ≥30 data from the WHO Global Health Observatory; GDP data from the World Bank; Urbanization data from WHO. Biological State Index (I_bs_) was self-calculated with country specific fertility data published by the United Nations and the mortality data published by World Health Organization (WHO). Gini index from the World Bank.
