# Supplementary material for: Relaxed natural selection contributes to global obesity increase more in males than in females due to more environmental modifications in female body mass
Source: PLoS One. 2018 Jul 18;13(7):e0199594. doi: 10.1371/journal.pone.0199594 (PMC6051589; doi:10.1371/journal.pone.0199594)
Supplement: S3 Table — (DOCX) [file pone.0199594.s003.docx]

**S3 Table: Tests of normality of distributions of studied variables**

|  | Kolmogorov-Smirnov^a^ | | |  | Shapiro-Wilk | | |
| --- | --- | --- | --- | --- | --- | --- | --- |
|  | Statistic | df | Sig. |  | Statistic | df | Sig. |
| BMI ≥ 30, Male | 0.138 | 168 | <0.001 |  | 0.933 | 168 | <0.001 |
| BMI ≥ 30, Female | 0.054 | 168 | 0.200^*^ |  | 0.984 | 168 | 0.052 |
| Caloric intake | 0.045 | 168 | 0.200^*^ |  | 0.981 | 168 | 0.023 |
| GDP | 0.249 | 168 | <0.001 |  | 0.683 | 168 | <0.001 |
| I_bs_ | 0.252 | 168 | <0.001 |  | 0.767 | 168 | <0.001 |
| Urbanization | 0.067 | 168 | 0.066 |  | 0.972 | 168 | 0.002 |
| *. This is a lower bound of the true significance.  a. Lilliefors Significance Correction  Sex specific obesity prevalence is the percentage of a defined population segment with a body mass index (BMI) of no less than 30 kg/m^2^.  Data sources: Total calories data from the FAO’s FAOSTAT; BMI ≥30 data from the WHO Global Health Observatory; GDP data from the World Bank; Urbanization data from WHO. Biological State Index (I_bs_) was self-calculated with country specific fertility data published by the United Nations and the mortality data published by World Health Organization (WHO). | | | | | | | |
