# Supplementary material for: Relaxed natural selection contributes to global obesity increase more in males than in females due to more environmental modifications in female body mass
Source: PLoS One. 2018 Jul 18;13(7):e0199594. doi: 10.1371/journal.pone.0199594 (PMC6051589; doi:10.1371/journal.pone.0199594)
Supplement: S4 Table — (DOCX) [file pone.0199594.s004.docx]

S4 Table: Correlation coefficients and Fisher’s r-to-z transformations of Pearson r and partial correlations between calories and female and male obesity prevalence

|  | Pearson Correlation  Calories | | | |  | Partial Correlation  Calories | | | | |
| --- | --- | --- | --- | --- | --- | --- | --- | --- | --- | --- |
| Variable | n | r | p | Fisher's r-to-z transformation |  | df | r | p | Effect Size | Fisher's r-to-z transformation |
| BMI 30, 18+M,2014 | 172 | 0.716 | 0.000 | z= 3.31 p=0.0005 |  | 163 | 0.259 | 0.000 | 0.072 | z=1.11  p=0.134 |
| BMI 30, 18+F,2014 | 172 | 0.493 | 0.000 |  |  | 163 | 0.140 | 0.073 | 0.020 |  |
| GDP 2010 USD | 168 | 0.759 | 0.000 | - |  | - | - | - | - | - |
| I_bs_ | 172 | 0.639 | 0.000 | - |  | - | - | - | - | - |
| Urbanization | 172 | 0.602 | 0.000 | - |  | - | - | - | - | - |
| Partial correlation (two-tailed) is reported. -, Controlled variable or not relevant.  BMI≥30 and BMI ≥ 25 are percentages of defined population with a body mass index (BMI) of no less than 30 kg/m2 and 25 kg/m2 respectively. Population fall in pre-obesity is indicated as “BMI 25-30, M”.  Data sources: Total calories data from the FAO’s FAOSTAT. BMI (≥30, ≥25 and mean) data from the WHO Global Health Observatory, and self-calculated pre-obesity (BMI≥25 but <30) by subtracting BMI ≥ 30 from BMI ≥25. GDP data from the World Bank. Urbanization data from WHO. | | | | | | | | | | |
