# Supplementary material for: Relaxed natural selection contributes to global obesity increase more in males than in females due to more environmental modifications in female body mass
Source: PLoS One. 2018 Jul 18;13(7):e0199594. doi: 10.1371/journal.pone.0199594 (PMC6051589; doi:10.1371/journal.pone.0199594)
Supplement: S1 Text — (DOCX) [file pone.0199594.s006.docx]

**S1 Text: Calculation and significance of Biological State Index (I_bs_)**

Natural selection is a key mechanism of evolution, the change in heritable traits of a population over successive generations [1]. In our modern society, natural selection still acts on all members of a population, selecting those individuals that have an increased reproductive success (survival and/or fertility) [2]. The “Biological State Index (I_bs_)” has been proposed to measure the populational reproductive success by taking into account potential loss of reproductive success by dying at age x [3, 4].

The I_bs_ is calculated by combining age-specific death frequency (d_x_ variable of a life table) with an age-specific reproductive loss (s_x_):

$$I_{bs}=1- \sum_{x=0}^{x=\omega} d_{x}.s_{x}$$

Where: d_x_ is the frequency of death at age x or represents the mortality rate. s_x_ is the reproductive loss from dying at age x, i.e. the estimated probability of not possessing the complete number of births at age x. s_x_ is based on the cumulative number of births at specific ages [4, 5]. The construction and interpretation of the I_bs_ was predicated upon the assumption that heritability of human fertility variance is negligible [6]. An I_bs_ value of one indicates total adaptation of the population to their environment (ability to overcome selection pressures that are present). An I_bs_ value of zero signifies a total lack of adaptation (inability to overcome selection pressures that are present), and an impossibility to give life to the next generation. An I_bs_ value close to zero indicates large effective natural selection pressures acting on a population, since few individuals are surviving to produce offspring. In such a scenario there is a possibility for fast evolution, since many genes may not be passed to the next generation. An I_bs_ value close to one indicates that natural selection does not have much effect on the population since many individuals are able to maximally contribute to producing the next generation. Thus, the I_bs_ permits the estimation of the magnitude of the successful reproduction of a population.

**References:**

1. Hall, B.K.H., Benedikt, *Strickberger's Evolution (4th ed.).* . 2008: Sudbury, MA: Jones and Bartlett Publishers. ISBN 978-0-7637-0066-9. LCCN 2007008981. OCLC 85814089.

2. Byars, S.G., et al., *Colloquium papers: Natural selection in a contemporary human population.* Proceedings of the National Academy of Sciences of the United States of America, 2010. **107 Suppl 1**: p. 1787-92.

3. Henneberg, M. and J. Piontek, *Biological state index of human groups.* Przeglad Anthropologiczny, 1975. **XLI**: p. 191-201.

4. Henneberg, M., *Reproductive possibilities and estimations of the biological dynamics of earlier human populations.* Journal of Human Evolution, 1976. **5**: p. 41-8.

5. Henneberg, M., *Notes on the reproduction possibilities of human prehistorical populations.* Przeglad Anthropologiczny, 1975. **41**: p. 75-89.

6. Henneberg, M., *Quantitative evaluation of actual intensity of natural selection through differential fertility in human populations,.* American Journal of Phys Anthropology, 1985. **66**: p. 181.
